# Zebrafish as an Integrative Model for Central Nervous System Research: Current Advances and Translational Perspectives

**DOI:** 10.3390/life15111751

**Published:** 2025-11-14

**Authors:** Lidia Pansera, Kamel Mhalhel, Mauro Cavallaro, Marialuisa Aragona, Rosaria Laurà, Maria Levanti, Maria Cristina Guerrera, Francesco Abbate, Antonino Germanà, Giuseppe Montalbano

**Affiliations:** 1 Department of Chemical, Biological, Pharmaceutical and Environmental Sciences, University of Messina, Viale Ferdinando Stagno D’Alcontres 31, 98166 Messina, Italy; lipansera@unime.it (L.P.); kmhalhel@unime.it (K.M.); 2 Zebrafish Neuromorphology Lab, Department of Veterinary Sciences, University of Messina, 98168 Messina, Italy; mcavallaro@unime.it (M.C.); mblevanti@unime.it (M.L.); mguerrera@unime.it (M.C.G.); abbatef@unime.it (F.A.); agermana@unime.it (A.G.); gmontalbano@unime.it (G.M.)

**Keywords:** central nervous system, neurological diseases, animal model, brain, zebrafish

## Abstract

Central nervous system disorders represent a heterogeneous set of conditions triggered by genetic alterations, environmental exposures, infections, injuries, and even iatrogenic causes. These conditions impact a significant portion of the global population, posing serious concerns for public health. Even though progress has been made in understanding and treating some of these disorders, many others remain poorly understood, with research still in their early stages. For that, adapted experimental models are essential for deciphering the physiopathology of disorders and developing future therapeutic strategies. Within this context, zebrafish (*Danio rerio*) has emerged as a valuable model for central nervous system disorders, thanks to its high genetic and neuroanatomical homology with humans, the conservation in different aspects of cellular architecture and blood–brain barrier, and the remarkable regenerative ability of the CNS. This review presents the state of the art on zebrafish models for central nervous system disorders, presenting their potential in comprehending the pathophysiological processes and screening therapeutics.

## 1. Introduction

Disorders affecting the central nervous system (CNS) represent a primary global health concern, as they are one of the leading causes of mortality and long-term disability. According to the World Health Report 2001, brain diseases account for 35% of total DALYs, standing for Disability-Adjusted Life Years, an indicator derived from years of life lost (YLL) due to early death and years lived with disability [1].

As global life expectancy continues to rise worldwide [1], the prevalence of age-related neurological disorders is projected to grow markedly [2]. Moreover, in addition to their profound social and health-related implications, CNS disorders have always exerted a significant economic impact on healthcare resources. Indeed, a study conducted between 2000 and 2019 across 204 countries, including 24 brain disorders, revealed a yearly 3.5% growth of direct healthcare spending [3]. This global report does not include research and development funding, which represents a minor fraction of overall health spending, although it has a significant impact on advancing scientific knowledge. Increasing CNS research funding seems to be less of a choice, as it is essential for ensuring continued progress in understanding disease mechanisms and developing efficacious prevention and treatment strategies. At this point, translational research using adapted animal models becomes critical. Among the most extensively employed models are murine species, which have significantly contributed to the breakdown of neurodegenerative diseases such as amyotrophic lateral sclerosis (ALS), Parkinson’s disease, Alzheimer’s disease, and Huntington’s disease, as well as psychiatric disorders and brain tumors [4,5,6,7]. Also, non-human primates have been used to provide a closer similarity of human brain anatomy and spinal cord function, particularly in the study of complex behaviors and higher cognitive functions [8,9]. Despite their translational relevance, their application is limited by high costs and poor availability. Moreover, other animal models such as pigs, sheep, and dogs are practical for imaging studies, surgical interventions, and preclinical evaluations of therapies, thanks to their anatomical and physiological features similar to those of humans [10,11,12,13,14]. Within this diverse landscape, the zebrafish (*Danio rerio*) has progressively consolidated its role as a key and complementary model, combining many of the advantages of higher vertebrate systems while minimizing their limitations. Indeed, zebrafish offer high genetic [15,16] and physiological homology [17,18,19] with humans, optical transparency of the embryos, rapid development [20], and large-scale screening and manipulations [21]. Owing to these features, this species serve as an effective model for investigating a wide range of disorders [22], including those affecting the central nervous system, and a powerful, cost-effective platform for translational neuroscience. Although zebrafish models are valuable for studying various aspects of CNS disorders, their use is hindered by several challenges, including the difficulty in assessing and reproducing complex behavioral and cognitive functions, as well as different pharmacokinetics and pharmacodynamics driven by a specie specific features of the blood–brain barrier (BBB). Such differences can interfere with the accurate interpretation and evaluation of the translational relevance highlighting the necessity of adopting a complementary approaches using other mammals models [23].

## 2. How Zebrafish Overcomes Key Limitations in CNS Research

### 2.1. Evolutionary and Biological Similarities with Humans

In 2013, the publication of the whole sequenced zebrafish genome [15] highlighted the extensive genetic similarity between this species and humans, fundamentally changing the perception of its potential. In fact, about 70% of human genes have at least one zebrafish orthologue, and more than 80% of genes associated with human disease are conserved [15].

Beyond this strong conservation, important genomic differences have also emerged. Teleost fish, including zebrafish, underwent an additional whole-genome duplication event (Ts3R) after diverging from the tetrapod lineage, which led to gene sub- or neo- functionalization and structural rearrangements characteristic of their genomes [24,25]. As a result of this evolutionary process, some human genes are represented by duplicated orthologues in zebrafish, whereas others are missing. For instance, the human amyloid precursor protein (APP) gene has two zebrafish orthologues, appa and appb, while the human α-synuclein (SNCA) orthologue is missing from the zebrafish genome [26,27,28].

**Figure 1:**
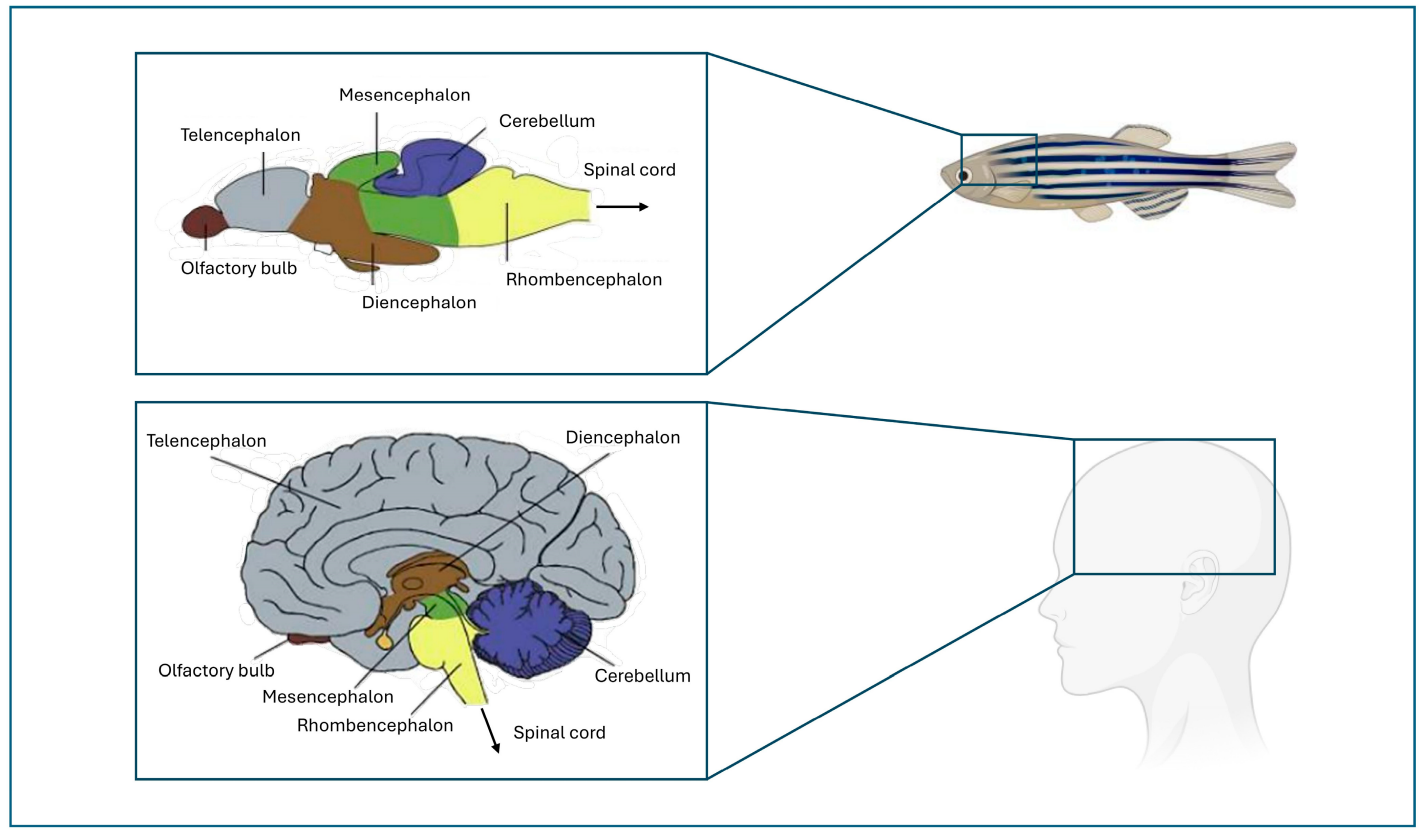
Comparative schematic representation of zebrafish and human brain regions highlighting the main homologous areas. Adapted from Ahmed et al. [48].

This combination of deep conservation and teleost-specific characteristics, makes the zebrafish genome both comparable to and distinct from the human one, providing a unique opportunity to investigate gene redundancy and functional divergence while modeling human diseases with a robust predictive value. At the neuroanatomical level, however, the development of the zebrafish telencephalon occurs by eversion, in contrast to the evagination observed in mammals [29]. This process influences the spatial arrangement of brain regions, ventricular organization, neuronal migration, and the orientation of neurogenic zones, ultimately leading to an inverted topography of telencephalic areas compared to mammals; however, the overall organization of the zebrafish central nervous system closely mirrors the mammalian one, maintaining the main divisions: forebrain (prosencephalon), midbrain (mesencephalon), and hindbrain (rhombencephalon), as shown in Figure 1. This tripartite structure is established early in development and becomes morphologically distinguishable by around one day post-fertilization (dpi) [30,31]. In zebrafish the forebrain comprises the telencephalon and the diencephalon. The telencephalon represent the most anterior part and is divided into dorsal and ventral regions, corresponding to the mammalian pallium and subpallium, respectively [32]. Within the dorsal telencephalon, distinct subregions exhibit functional similarities to mammalian brain structures: The dorsomedial zone (Dm) shares structural and functional features with the amygdala, mediating emotional learning and fear responses; the central zone (Dc) corresponds to the cerebral cortex, integrating sensory input and participating in higher-order processing; the dorsolateral and dorsoposterior areas (Dl/Dp) are considered as hippocampal homologous, and play key roles in spatial learning and memory [33,34]. The diencephalon, including the thalamus, hypothalamus, and epithalamus, retains conserved roles in sensory relay, homeostatic regulation, and circadian rhythms. The zebrafish hypothalamus is of particular interest, as it contains neurosecretory systems and nuclei involved in stress response, metabolism, and neuroendocrine signaling, highly conserved and functionally significant in vertebrate neuroendocrinology [35,36]. The mesencephalon (midbrain) encompasses structures analogous to the mammalian tectum (superior colliculus) and tegmentum, essential for visual processing, sensorimotor integration, spatial orientation, and locomotor control [37]. Both tectum and tegmentum in zebrafish participate in transforming visual information into behaviors such as prey capture, escape, and orienting movements [38]. These areas contribute to control movements and reflexive responses, supporting their use in visual and motor circuitry studies [39]. The hindbrain includes the cerebellum (dorsal anterior hindbrain), medulla oblongata (caudal hindbrain), and pons-like regions that, although less anatomically defined than in mammals, serve comparable functions [40]. This zone plays a central role in maintaining balance, autonomic regulation, and learning of motor skills [41]. The zebrafish cerebellum, despite its simpler structure, shares developmental origin, cellular architecture (molecular layer, Purkinje cell layer, and granule cell layer) [42], and molecular markers with its mammalian counterpart [43]. However, it also exhibits teleost-specific features, including distinct subdivisions such as the valvula cerebelli and the corpus cerebelli [44], absent in mammals. Moreover, the zebrafish cerebellum contains intrinsic cerebellar nuclei that contribute to species-specific patterns of connectivity and signal integration [42,45]. Furthermore, zebrafish share all major neurotransmitter systems with mammals, including dopaminergic [42], serotonergic [43], GABAergic [46], and glutamatergic signaling pathways [47]. These signaling networks are fundamental to neuronal communication, regulating key processes such as neurodevelopment, synaptic plasticity, cognition, and behaviour [46]. These strong similarities to mammals reflect deep evolutionary conservation in terms of synthesis, transport, and receptor function.

### 2.2. Modelling Disease Complexity

**Figure 2:**
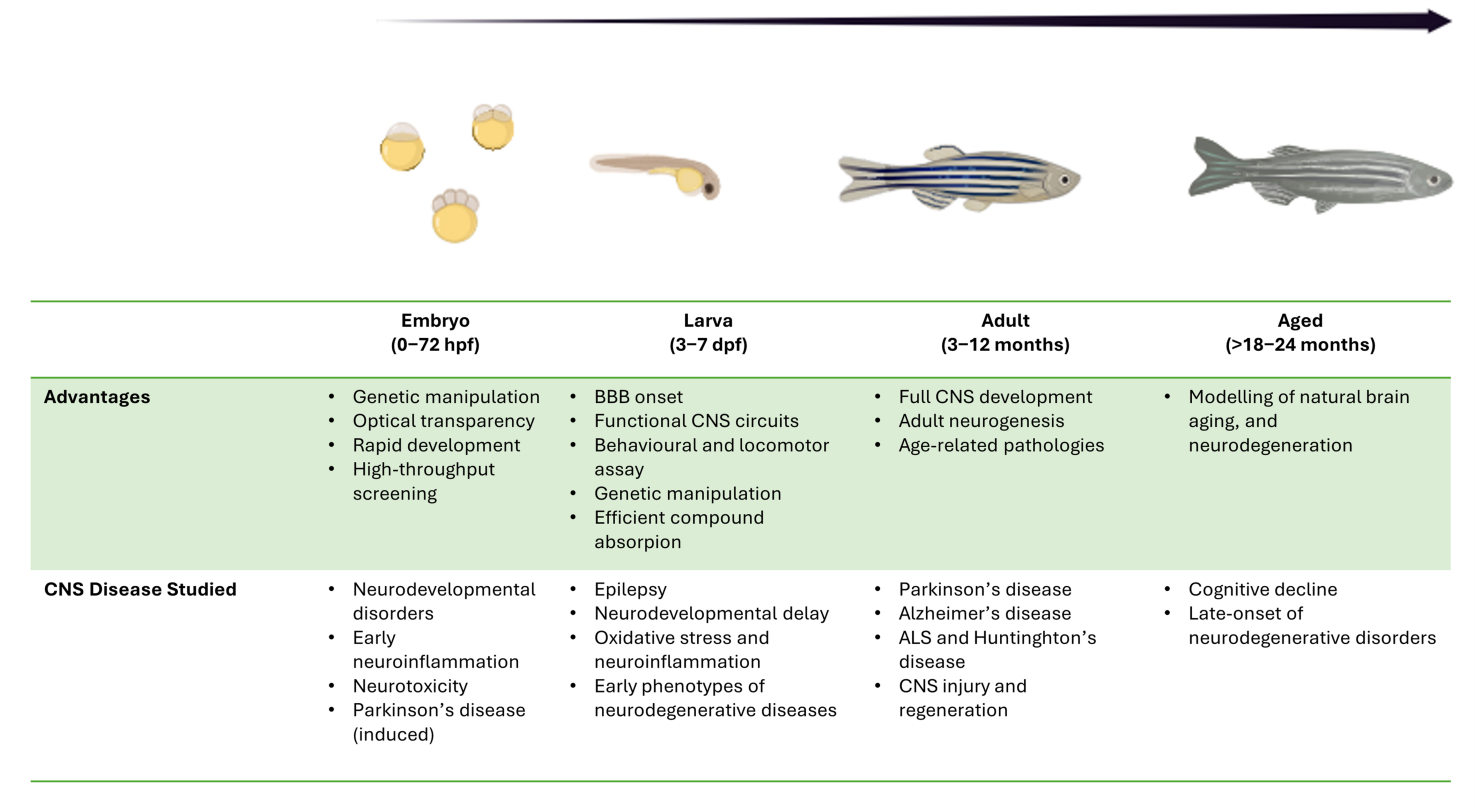
Advantages and CNS diseases investigated across zebrafish (*Danio rerio*) developmental stages.

Zebrafish is a relatively small diploid vertebrate able to support a diversity of experimental approaches, reproducing complex pathological phenotypes (Figure 2). In fact, they share with humans remarkable similarity in key biological mechanisms, covering hematopoiesis pathways and some regulatory processes including inflammation and immune cell development [49,50]. These processes are deeply involved in the onset and progression of numerous diseases, including neurodegenerative disorders [51,52].

Another strength point of this system is that researchers can induce disease-like conditions using chemical exposure, surgical or mechanical injuries, and environmental perturbations, thereby modelling CNS disease and reproducing key pathological features such as dopaminergic neuron loss, motor dysfunction, and oxidative stress [53,54,55]. Similarly, targeted injuries, including spinal cord transection or traumatic brain injury, provide in vivo models to investigate tissue damage, inflammatory responses, axonal regrowth, neurogenesis, and glial bridging [56,57,58]. Thus, zebrafish offer a unique opportunity to investigate the cellular and molecular processes underlying central nervous system (CNS) disorders even though they exhibit both conserved and distinct features of the process compared to mammals. In zebrafish the innate immune system matures first during development, with macrophages appearing as early as 15 hpf and becoming capable of phagocytosis, reactive oxygen species (ROS) production, and pathogen elimination around 26 hpf [59,60]. Neutrophils appear around 18 hpf and reach maturity between 24 and 48 hpf, that share homologous molecular markers and functions with their mammalian counterparts, such as phagocytosis, cytokine secretion, and reactive oxygen species production [61,62]. Their adaptive immune system, comprising T- and B-cell maturation becomes functional at later development stage (after 3–4 weeks post-fertilization), providing a temporal window that allows to analyze the innate immune response independently [63,64]. Moreover, certain cytokine gene families are duplicated or divergent due to teleost-specific genome duplication [65]. Still, key proinflammatory pathways including TNF-α, IL-1β, IL-6, and NF-κB are functionally conserved in zebrafish, enabling real-time visualization of inflammatory dynamics [66,67]. The same is true for the blood–brain barrier, which shows architectural features reflected by differential permeability, treated in the following section. These distinctions must be considered when interpreting neuroinflammatory outcomes.

Additionally, beyond single-factor models, zebrafish enable the multifactorial nature of many neurological diseases by combining different risk components. For instance, co-exposure to aluminum chloride and D-galactose has been reported to induce Alzheimer’s-like phenotypes, characterized by amyloid accumulation, oxidative stress, and cognitive impairment, thereby integrating metal toxicity with ageing-related metabolic alterations [68]. These results highlight how the combination of distinct stimuli can synergistically intensify neuropathology outcomes. Environmental influences can also modulate later disease susceptibility. It is known that zebrafish embryos exposed to ethanol show a markedly increased sensitivity to pentylenetetrazole (PTZ)-induced seizures, demonstrating how prenatal environmental factors can prime the nervous system for heightened epileptogenic responses [69].

### 2.3. Overcoming BBB and Pharmacokinetic Barriers

The blood–brain barrier (BBB) is a highly specialized interface formed by endothelial cells lining the brain microvessels. Its primary role is to tightly regulate the exchange of substances between the circulation and the central nervous system, thereby preserving the CNS from neurotoxins, pathogens, and fluctuations in blood composition [70,71], maintaining brain homeostasis required for proper neural function [72] and playing a fundamental role in defense. The neurovascular unit (NVU), the basic structural unit of the BBB, is made of cerebral vascular endothelium, neurons, pericytes, astrocytes, and the extracellular matrix, alongside crucial components of brain microenvironment namely microglia, macrophages, and fibroblasts. The links between the components of the NVU insure a modulation of the BBB in response to stimuli [73]. In mammals tight junctions linking the endothelial cells, limit paracellular diffusion, and insure a selective transport, controlling the movement of ions, nutrients, and signaling molecules into the brain [74]. Tight junction transmembrane proteins contributing to the BBB are lipolysis-stimulated protein, occludin, claudin-1, -3, -12, and the most abundant claudin-5 [75]. These tight junctions are mainly linked to actin cytoskeleton by intracellular scaffold proteins, the zonula occludens (ZO) [75].

Pericytes, the principal component of a canonical NVU, embedded in the vascular basement membrane, are required for the formation and maintenance of the BBB, forming cell–cell contacts with endothelial cells and astrocytic end-feet [76,77]. Astrocytes, however, form contacts with both microvessel walls and neurons, and they are important in the modulation and maintenance of the BBB [73].

The zebrafish BBB shows extensive structural and molecular conservation with mammals. Brain endothelial cells form tight junctions with high electrical resistance (such as claudin-5 and ZO-1), exhibit low levels of transcytosis, lack fenestrations, and express nutrient transporters such as Glut1 (Slc2a1). Still, it lacks the typical mammalian astrocytes, which role have been covered by radial glial cells [78]. These features collectively confer selective permeability comparable to the mammalian barrier [79,80]. The barrier is also embedded within a conserved neurovascular unit (NVU) [81], where pericytes and glia cells interact with endothelium. Based on several studies, the zebrafish blood–brain barrier (BBB) has a lower pericyte density and a thinner basal lamina when compared to mammals [82,83]. The zebrafish pericyte, detectable from ~60 hpf, mirror mammalian pericytes’ role [84,85]. Functionally, permeability assays indicate that the first signs of a functional BBB appear around ~2.5 dpf and become more established by ~3 dpf [83]. Moreover, although key tight junction proteins like claudin-5, occludin, and ZO-1 are conserved between zebrafish and humans they are fewer and less compact [79]. Do the zebrafish BBB increased permeability, allowing the passage of hydrophilic substances and xenobiotics that would be blocked by a mature mammalian BBB [83,86]. These conserved elements make zebrafish an advantageous model for investigating BBB formation and maturation [81,87]. It is also helpful in assessing how drugs and other molecules cross the barrier in vivo, as zebrafish BBB permeability correlates well with data from mouse studies, supporting its translational relevance [88].

### 2.4. Assessing Behavioural and Cognitive Outcomes

Behavioral and cognitive phenotyping is a central component of zebrafish research, providing functional endpoints that complement molecular, histological, and physiological analyses. Since many neurological, neurodegenerative, and psychiatric disorders manifest as changes in locomotor, learning, or social interaction, the ability to assess these outcomes in vivo is essential to understanding disease mechanisms and evaluating potential therapeutic strategies. Thanks to its conserved neuroanatomy and neurochemical signaling, zebrafish is widely employed to model human-relevant cognitive functions and behavioral alterations [89,90,91]. Behavioral assessments can be used for both simple sensorimotor tests to more complex paradigms evaluating cognitive performances. Still this crediting depends on the developmental stage (Table 1). Most paradigms can be reliably applied from the early larval phase (5–7 dpf), when spontaneous swimming, startle reflexes, phototaxis, and thigmotaxis are already established and quantifiable [92]. In contrast, more complex cognitive and social tests require a higher degree of neural development [93,94]. Anxiety-like behaviors in zebrafish are commonly evaluated using assays adapted from rodent models, such as the novel tank diving test, which measures exploratory drive through changes in vertical positioning, or the open field test, which evaluates locomotor activity and thigmotaxis [95]. Olfactory-based approaches like the novel odour exploration test (NOEt) further exploit the species’ chemosensory abilities to reveal anxiety-related responses [96]. Cognitive functions are assessed through tasks probing memory and learning: the T-maze paradigms test spatial navigation, the novel object recognition (NOR) test evaluates memory by measuring preference for novelty, and conditioned place preference (CPP) explores associative learning and reward sensitivity [96,97,98]. At a more advanced level, behavioral analyses include prepulse inhibition (PPI), a key index of sensorimotor gating used to model schizophrenia and related disorders, as well as social interaction assays, including shoaling and conspecific recognition [99]. At the larval stage, behavioral assays are particularly valuable because they can be performed in a high-throughput manner using large sample sizes. Larvae already display stereotyped and quantifiable behaviors within days after fertilization [100,101]. Underlying these behavioral outputs are neurotransmitter systems remarkably conserved with mammals, enabling the investigation of the functional state of dopaminergic, serotonergic, and glutamatergic circuits [102]. The advent of high-content behavioral platforms and automated tracking systems has substantially improved the precision and reproducibility of these assays, facilitating the identification of phenotypic changes caused by neurotoxic insults, genetic mutations, or drug treatments [103]. Behavioral assessment also represents a crucial functional endpoint in preclinical research, especially when evaluating the efficacy, safety, or potential side effects of pharmacological agents and bioactive compounds. Since many neuroactive substances exert their effects modulating neuronal activity, neurotransmission, or synaptic plasticity, changes in locomotion, anxiety-like responses, learning capacity, or social interaction can provide sensitive and early indicators of therapeutic potential [104]. The development of advanced automated tracking platforms has further enhanced this field by enabling high-resolution, high-throughput behavioural monitoring under controlled conditions. Despite these advantages, interpreting zebrafish behavior requires careful experimental design and validation, as variables such as age, sex, circadian rhythm, and environmental conditions can influence the outcomes, making standardized protocols essential for reproducibility [101]. Moreover, while zebrafish behaviors are often analogous to those of mammals, they are not identical. Indeed, several limitations hinder the cross-species comparison and the direct extrapolation of behavioral data from zebrafish to mammals. Although they express genes homologous to those implicated in cortical malformations clinically, zebrafish lack a laminated neocortex, the cortical component of the mammalian telencephalon responsible of volitional motor control, perception, cognition and other complex behaviors [105,106]. Moreover, the zebrafish telencephalon consists mainly of regions homologous to the mammalian cortex, hippocampus, and some sections of the amygdala [33], which, although functionally analogous, differ markedly in cytoarchitecture and connectivity constraining the interpretation of complex cognitive and emotional behaviors [107,108,109]. Finally, standardization issues, including variation in experimental conditions, developmental stages, and behavioral endpoints, complicate cross-species comparisons. Taken together, these considerations highlight that, although zebrafish share fundamental behavioral and neurochemical mechanisms with mammals, interpretation of behavioral testing requires careful contextualization, ideally supported by complementary mammalian models. Indeed, as explained by Gerlai (2020) zebrafish definitely can’t recapitulate all aspects of human memory, behavioral, neuroanatomical, and molecular phenomena, but it could guide researchers to the evolutionarily conserved and thus the most important features and mechanisms, which integrated with complementary approaches using other mammals, could results in discoveries that are highly translational [23].

### 2.5. Aligning with Ethical Principles and 3Rs

The use of animals in scientific research is regulated by an ethical and legal framework designed to ensure both animal welfare and high scientific quality. In the European Union, the primary reference is Directive 2010/63/EU on the protection of animals used for scientific purposes [116], which establishes legally binding standards for all vertebrate models, including zebrafish (*Danio rerio*). Under the Directive, animal use must be scientifically justified, procedures causing pain, suffering, distress, or lasting effects must be minimized, and projects must undergo ethical review and authorization by competent authorities before they begin. Central to this legislation is the principle of the 3Rs (Replacement, Reduction, and Refinement) first formulated by Russell and Burch in 1959 [117]. A particularly relevant aspect for zebrafish research is the provision in Directive 2010/63/EU and subsequent amendments, particularly the Commission Implementing Decision 2020/569/EU of 16 April 2020 (Anne III, Part B, B, 1.2), and repealing the Implementing decision 2012/707/EU, that clarified that zebrafish embryos and larvae up to the stage of independent feeding (5 dpf) are not considered as protected animals under EU legislation. During these early developmental stages, withdrawal behaviors are present. However, in the absence of mature forebrain structures, these reflexes are unlikely to be accompanied by an emotional experience of pain [108]. For this reason, experiments conducted before 120 hpf do not require specific project authorization or ethical approval, provided that welfare standards are respected [116]. Regulation further strengthened the ethical landscape (EU) 2019/1010, which updated aspects of Directive 2010/63/EU to improve transparency, reporting, and harmonization of data on animal use [116]. The exemption for early zebrafish stages is particularly valuable in pharmacological and toxicological research, where large-scale compound screening and dose–response studies are essential. Between 2008 and 2012, the European Union Reference Laboratory for Alternatives to Animal Testing (ECVAM) has led the multi-laboratory validation of the zebrafish embryo acute toxicity test (ZFET), and supported the validation reports that were used to develop OECD TG 236 [118]. This protocol allows the assessment of teratogenicity, cardiotoxicity, neurotoxicity, and developmental toxicity without the need of animal experimentation authorization [119]. When performed within this developmental window, behavioral and drug efficacy assays provide rapid and ethically compliant insights into compound activity, helping researchers to screen promising candidates before advancing to complex in vivo models. In this way, zebrafish research is strongly aligned with the 3Rs principles, demonstrating how regulatory compliance, ethical responsibility, and scientific innovation can effectively coexist to advance biomedical science.

## 3. Zebrafish Models of CNS Disorders

Zebrafish represents a highly versatile vertebrate model that allows the reproduction of diverse pathological mechanisms observed in human CNS diseases. Different experimental paradigms have been established, including toxin exposure, physical injury, and genetical manipulation, each offering complementary insights into neurodegeneration and regeneration. To provide an overview, Table 2 summarizes representative zebrafish models and the respective human diseases.

### 3.1. Spinal Cord Injury (SCI): Transection, Crush, Laser Ablation Models

Spinal cord injury (SCI) is among the most severe conditions affecting the central nervous system. It often leads to long-lasting motor and sensory impairments, mainly because of the limited ability of mammalian neurons to regenerate after damage. In contrast, the zebrafish (*Danio rerio*), exhibits a remarkable ability to regenerate spinal tissue, making them a powerful vertebrate model for dissecting the mechanisms underlying neural repair and for developing novel therapeutic strategies. To replicate SCI, different experimental paradigms, including transection, crush, and laser ablation, have been developed, each offering distinct levels of injury severity, reproducibility, and translational relevance. Transection models involve the complete severing of the spinal cord, generating a reproducible and well-defined lesion with a clear boundary between damaged and intact tissue. The lesion is typically induced using micro-needles, tungsten micro-needles, or sharpened glass micropipettes. These models are widely applied to explore axonal regrowth, glial bridging, and functional recovery [132]. Remarkably, adult zebrafish can recover coordinated swimming within 6–8 weeks after complete transection, accompanied by robust neuronal regeneration and synaptic reconnection [148,149]. Transection paradigms have also served as platforms for testing therapeutic approaches, including anti-inflammatory drugs and neurotrophic factors, which significantly enhance regenerative outcomes [133]. Crush injury models offer a physiologically relevant alternative that closely mimics the partial mechanical compression seen in clinical SCI. These injuries are induced by applying controlled pressure to the exposed spinal cord using calibrated forceps, tungsten needles, or micro-needles, producing axonal damage without complete severing [134]. This approach allows the investigation of demyelination, axonal sprouting, neuroinflammatory responses, and glial cell activation. Adult zebrafish show rapid regenerative responses following crush injuries, including microglial activation, glial bridge formation, and axon extension within days [135,150]. Crush models are suitable for evaluating the effects of candidate molecules or key genes identification on regeneration dynamics, offering valuable insights into potential therapeutic targets [135,136,151]. Indeed, in a study conducted by Hui et al. (2010) [135], the spinal cord was crushed dorso-ventraly for 1 sec with a forceps. This study characterized the injury-induced cellular responses in adult zebrafish spinal cord highlighted mainly by the infiltrations of blood cells during early phases, probably implicated in suppressing inflammatory response, as well as the recruitment of radial glia as proliferating precursor in neurogenesis [135]. Moreover, the study by Zeng et al. (2021) [151] identified a new population of injury-induced cells in a zebrafish embryos SCI model, called stress-responsive regenerating cells (SrRCs), which appear in both the rostral and caudal sides. Still, the rostral SrRCs regenerated neurons more effectively than caudal-side ones. This efficient regeneration was correlated to higher expression of caveolin-1, which promotes axonal regrowth and bridge formation across the injury [151]. Within the same context, Shen et al. (2022) analyzed gene expression during the subacute phase by which adult zebrafish regained about 44% of their swimming ability and identified 7762 differentially expressed genes, part of which were involved in axon regeneration and neural differentiation, including CLASP2, which promotes microtubule stabilization and axonal extension, and H1M, which regulates neural stem cell differentiation [136]. Laser ablation models represent the most precise and minimally invasive SCI paradigm, especially effective in larval and juvenile zebrafish. Using targeted high-energy laser pulses, researchers can ablate specific regions of the spinal cord or even individual neuronal populations [152]. This enables highly controlled, cell-type-specific injury and fine-scale analysis of regenerative processes. The optical transparency of zebrafish larvae allows real-time imaging of cellular dynamics, including immune cell infiltration, axonal regrowth, and synaptic remodeling, during degeneration and repair.

### 3.2. Traumatic Brain Injury (TBI)

Traumatic brain injury (TBI) is a major cause of neurological disability worldwide, often resulting in long-lasting motor, cognitive, and behavioral impairments due to the limited regenerative potential of the mammalian central nervous system. Among the various experimental approaches, the stab injury model is one of the most widely used paradigms to mimic focal traumatic lesions in zebrafish, allowing precise control of injury severity, spatial localization, and reproducibility. To perform this technique, a fine surgical instrument, typically a tungsten micro-needle, micro-scalpel, or sharpened glass capillary, is carefully inserted into a defined region of the brain, most often the telencephalon. This controlled lesion induces localized primary events, including neuronal death, axonal disruption and degeneration, and loss of synaptic connections. The following secondary phase is characterized by a controlled inflammation, and the reactivation of developmental signaling pathways, including Wnt/β-catenin, Notch, FGF, and Shh, thus enhances proliferation and differentiation [58,153]. This process is supported by a permissive microenvironment characterized by limited glial scarring. Radial glia and neural progenitor cells in the ventricular zone are rapidly activated, proliferating and migrating to the injury site, where they differentiate into new neurons and glial cells that integrate functionally into existing circuits [137,154,155]. In contrast, mammalian brains respond to trauma with extensive astroglial scar formation, mainly induced by reactive astrocytes, pericytes, infiltrating fibroblasts, and Schwann cells gather at the lesion site inhibitory extracellular matrix deposition; and chronic infiltration of inflammatory cells from both central and peripheral compartments [156], all of which hinder neurogenesis and axonal regeneration [157,158,159,160,161]. Thus, the scar formation thought to create both physical and chemical barriers that impede axon regrowth upon trauma [153].

Zebrafish stab injury model displays a robust regenerative response that is largely absent in mammals. The superior regenerative ability of zebrafish could be explained by the persistence of widespread neural stem and progenitor cells throughout the adult brain [162,163].

Moreover, the reproducibility of the zebrafish stab model makes it particularly suitable for studying the temporal dynamics of neurodegeneration, gliosis, neurogenesis, and functional repair. The stab model has been instrumental in identifying key molecular pathways involved in regeneration, inflammatory signaling cascades, and the neuroimmune response to trauma [138]. From a translational perspective, the zebrafish stab injury model is increasingly applied in drug screening and therapeutic testing [164].

### 3.3. Neurodegenerative Disorders

#### 3.3.1. Alzheimer’s Disease

Alzheimer’s disease (AD) is the most widespread neurodegenerative disorder worldwide. The main alterations involve the abnormal accumulation of proteins, particularly extracellular amyloid-beta (Aβ) plaques and intracellular tangles of hyperphosphorylated Tau, that lead to synaptic dysfunction, neuronal loss, and progressive neurodegeneration, cognitive decline and impairment of brain functions [165].

These main pathological hallmarks have been successfully modelled in zebrafish (*Danio rerio*), allowing in vivo observation of Aβ plaque formation, pathological Tau accumulation, and the associated neuronal alterations [166]. The zebrafish AD models are developed either through genetic modifications, introducing human genes mutated in Alzheimer’s disease, or by exposure to neurotoxic agents such as Aβ oligomers [167,168]. One of the most common approaches is the direct microinjection of synthetic human Aβ_1–42_ peptides into the zebrafish brain ventricles or directly into brain tissue of zebrafish larvae, that induces neuronal apoptosis, oxidative stress, and memory impairment similar to human AD [122,169]. Other studies have subjected zebrafish to prolonged exposure to Aβ oligomers dissolved in water, thereby modeling progressive and chronic neurotoxicity [170]. These models display cognitive and motor deficits, increased production of reactive oxygen species (ROS) in the brain, and disturbances in neuronal metabolism [27,139]. Complementary insights come from genetic modeling. Previous studies have demonstrated that overexpression of human APP or PSEN1 mutations reproduced amyloid accumulation, enabling long-term studies on plaque formation and therapeutic screening [139,168]. In addition, Tau-based models extend the Aβ framework by reproducing the intracellular pathology of AD. In zebrafish, the overexpression of human wild-type or mutant Tau results in hyperphosphorylation, cytoskeletal disruption, aggregation into neurofibrillary tangle, and subsequent neuronal death [146,171]. Moreover, transgenic zebrafish expressing human MAPT (microtubule-associated protein tau) have been developed to visualize tauopathy-related processes in vivo [146]. This model rapidly recapitulates key pathological features of tauopathies, such as hyperphosphorylation and conformational changes in tau protein, formation of neurofibrillary tangles, neuronal dysfunction, and cell death, all observable within the transparent zebrafish larvae [172]. Thanks to advanced imaging techniques and the specific features of the zebrafish model, it is also possible to perform time-lapse imaging to track tau aggregation and neuronal degeneration dynamically over time [146]. This enables visualization not only of the spatial and temporal propagation of tau pathology but also of associated cellular alterations like synaptic dysfunction and neuronal death at cellular and subcellular resolution, something that is challenging to achieve in mammalian models.

Decorticating these physiopathological processes provides a valuable framework for investigating the molecular mechanisms underlying tau hyperphosphorylation, including kinase-mediated regulation and axonal transport disruption, as well as for screening kinase inhibitors and aggregation modulators, contributing to the identification of potential therapeutic strategies. The combination of Aβ and Tau models has greatly advanced understanding of the synergistic interplay between these two pathologies and their contribution to neurodegeneration. Furthermore, zebrafish behavioural assays, including learning and memory tests, complement the characterization of zebrafish AD models, allowing functional evaluation of cognitive decline and recovery following pharmacological intervention.

#### 3.3.2. Parkinson’s Disease

Parkinson’s disease is a pathological syndrome involving multiple brain regions, characterized by progressive asymmetric slowness of movement, rigidity, and tremor, associated with neuronal loss and the formation of α-synuclein-containing proteinaceous aggregates in neurons of the substantia nigra [173]. Parkinsonism might have diverse pathological underlying causes, including tau, polyglutamine, and Alzheimer’s disease pathology, as well as nigral cell loss without hallmark pathological features [173,174]. Moreover, Parkinson’s disease seems to have a substantial genetic and environmental component. Indeed, three well validated autosomal dominant genes (SNCA, LRRK2, and VPS35) and three well validated autosomal recessive genes PRKN, PINK1, and DJ1 are known to cause Parkinson’s disease [173,175], inducing defects in mitochondria, axonal transport, and the dopaminergic system [176,177]. Zebrafish offer complementary toxin-based and genetic Parkinson’s Disease (PD) approaches that reproduce many of the key dopaminergic dysfunction and motor phenotypes. PD biochemical and behavioral features including a reduced tyrosine hydroxylase (TH) expression, catecholamine depletion, and both motor and non-motor symptoms induction using 1-methyl-4-phenyl-1,2,3,6-tetrahydropyridine (MPTP) was largely studied in adult zebrafish [120]. Other commonly employed neurotoxins, including 6-hydroxydopamine (6-OHDA), rotenone, and paraquat, produce dopaminergic vulnerability through oxidative and mitochondrial stress pathways-induction [121]. Critical reviews have provided detailed comparative evaluations of these toxin-based model phenotypes, evaluating both larval and adult stages, and how they complement mammalian models in terms of reproducibility and costs [54]. Genetic studies have further advanced the use of zebrafish in PD research by enabling the investigation of disease mechanisms, gene-environment interactions, and molecular pathways underlying neuronal degeneration. Similarly, transgenic zebrafish lines that overexpress or carry knock-in variants of human α-synuclein (SNCA) exhibit progressive protein aggregation and dopaminergic neurodegeneration, providing a valuable in vivo platform to investigate α-synuclein-driven mechanisms [178]. Nevertheless, despite their numerous advantages, zebrafish models of Parkinson’s disease present some limitations that should be considered when translating findings to humans, including their non-authentic Lewy bodies and their quite simplified dopaminergic system compared to that of mammals [179,180].

### 3.4. Chemically Induced Neurotoxicity

Chemically induced neurotoxicity models in zebrafish (*Danio rerio*) have become fundamental tools for investigating how environmental contaminants and xenobiotics contribute to neuronal damage, neurodevelopmental disorders, and neurodegenerative diseases. This species expresses functional orthologues of human P450 (CYP) families (CYP1A, CYP2, and CYP3), displaying comparable catalytic activity and substrate specificity [181]. CYP are a superfamily of the major mammalian cytochrome hemoproteins which largely participate in the oxidative metabolism of xenobiotics [182,183]. Through this conserved enzymatic network, zebrafish can metabolize various classes of substances, such as pharmacological agents and environment contaminants, providing a realistic metabolic context for assessing compound stability, clearance, and neurotoxic potential [184,185]. Among the most widely studied neurotoxicants are pesticides, particularly organophosphates (OPs) such as chlorpyrifos (CPF), diazinon, and malathion [186,187]. These compounds exert their toxicity mainly through irreversible inhibition of acetylcholinesterase (AChE). When this enzyme is inhibited, acetylcholine accumulates at synapses, keeping neurons in a persistent cholinergic overstimulation, and eventual neuronal death [188]. Zebrafish larvae exposed to CPF show pronounced behavioral alterations, including hypo or hyperlocomotion, impaired escape responses, and learning and memory deficits [90,123]. Beyond acute neurotoxicity, sublethal and chronic exposures disrupt synaptogenesis, neuroendocrine regulation, and dopaminergic signaling, mirroring neurodevelopmental impairments reported in humans [124]. CPF exposure has also been linked to increased reactive oxygen species (ROS), mitochondrial dysfunction, and apoptotic pathway activation [124]. These findings highlight zebrafish as a sensitive vertebrate model for understanding both the acute and long-term neurotoxic effects of organophosphates. Heavy metals represent another major class of neurotoxic pollutants with profound effects on the central nervous system. Mercury (Hg), particularly in the form of methylmercury, can cross the blood–brain barrier and accumulate in neural tissue, where it interferes with neuronal precursor proliferation, impairs synaptic development, and induces oxidative stress, ultimately leading to cognitive and motor deficits [128]. Lead (Pb) disrupts calcium-dependent signaling, and interferes with synaptic vesicle release, altering neuronal excitability, and leading to changes in movement patterns, learning ability, and anxiety-like behaviours in zebrafish larvae [129]. Similarly, cadmium (Cd) exposure causes apoptosis, mitochondrial damage, and chronic neuroinflammation, even at sublethal concentrations, and has been linked to abnormal neurodevelopment and behavioural alterations [130]. Importantly, combined exposures to multiple metals often result in synergistic neurotoxic effects, reflecting the real-world environmental conditions more accurately than single-compound experiments [131].

### 3.5. Inflammatory and Oxidative Stress Models

#### LPS-Induced Systemic Inflammation

Inflammation and oxidative pathways are deeply interconnected to drive the onset and progression of numerous neurodegenerative diseases, including Alzheimer’s, Parkinson’s, amyotrophic lateral sclerosis (ALS), and multiple sclerosis [189]. These processes contribute to neuronal death, synaptic dysfunction, mitochondrial damage, and impaired neuroplasticity. A widely used approach to induce neuroinflammation in zebrafish is through exposure to bacterial endotoxins such as lipopolysaccharide (LPS), a well-known activator of the innate immune response [118]. LPS binds to Toll-like receptor 4 (TLR4), triggering microglia and astroglia activation and the subsequent release of pro-inflammatory cytokines such as IL-1β, TNF-α, and IL-6 [126]. After exposure to LPS, zebrafish exhibit marked glial activation, leukocyte infiltration, oxidative burst, and behavioral alterations, including locomotor deficits and anxiety-like responses, which closely mirror the neuroinflammatory cascades observed in mammalian brains [127]. These models are crucial for investigating how chronic inflammation contributes to neurodegeneration and for testing the efficacy of anti-inflammatory agents and immunomodulatory compounds. Oxidative stress models, on the other hand, exploit the high sensitivity of neurons to reactive oxygen species (ROS) and reactive nitrogen species (RNS), which can damage proteins, lipids, and DNA, ultimately leading to neuronal apoptosis [163,190]. These oxidative insults also activate redox-sensitive signaling pathways, including Nrf2/ARE, MAPK, and NF-κB, which regulate antioxidant defenses and inflammatory responses [191,192]. Genetic manipulation of oxidative stress regulators, such as sod1, nos2, and nrf2, further complements pharmacological approaches and allows detailed mechanistic dissection of oxidative damage pathways. At present, most research in this field is focused on preventing or mitigating neuroinflammation and oxidative stress before irreversible neuronal damage occurs. This includes testing anti-inflammatory drugs that block cytokine production or microglial activation, as well as antioxidant molecules, both synthetic and natural, that scavenge free radicals, enhance endogenous antioxidant capacity, or activate protective pathways such as Nrf2. Such preventive approaches are particularly promising, as they aim not only to slow disease progression but also to address the early events that trigger the neurodegenerative process.

### 3.6. Transgenic Lines

Transgenic zebrafish lines have become one of the most powerful tools in modern neuroscience, allowing researchers to model human neurological diseases with precision and versatility. Owing to their high genetic homology with humans, rapid development, and suitability for target genetic manipulation, zebrafish provide a unique vertebrate system to investigate the molecular basis of neurodegeneration, neural circuit dynamics, and therapeutic interventions in vivo. Transgenic lines are typically generated by integrating exogenous DNA constructs into the zebrafish genome through techniques such as Tol2 transposon-mediated insertion, CRISPR/Cas9 knock-in, or BAC transgenesis [193,194]. These approaches allow targeted expression of human disease genes, fluorescent reporters, or regulatory elements under tissue-specific promoters. This versatility has led to the development of various zebrafish models that faithfully recapitulate key aspects of central nervous system (CNS) pathologies, from protein aggregation and synaptic dysfunction to neuroinflammation and neuronal death [195]. In Alzheimer’s disease research, for example, transgenic lines overexpressing human APP, PSEN1, or MAPT (Tau) have been developed to study amyloid plaque formation, Tau hyperphosphorylation, and their downstream effects on neuronal viability and cognitive function [146]. Similarly, transgenic models of Parkinson’s disease carrying human SNCA (α-synuclein) or mutations in PINK1, Parkin, or LRRK2 reproduce the selective loss of dopaminergic neuron, mitochondrial dysfunction, and behavioral deficits, offering valuable platforms for drug discovery and mechanistic studies [54,143,145,179]. In the context of amyotrophic lateral sclerosis (ALS), lines expressing mutant forms of SOD1, TDP-43, or FUS recapitulate motor neuron degeneration and neuromuscular junction damage, allowing real-time imaging of disease progression and therapeutic testing [140]. Collectively, these transgenic approaches have generated a wide array of zebrafish models for CNS disorders, ranging from Alzheimer’s disease to ALS and frontotemporal dementia. In addition to disease modeling, transgenic lines expressing fluorescent reporters under glial, neuronal, or synaptic promoters enable visualization of neurobiological processes such as neurogenesis, axonal regeneration, and neuroinflammatory responses [196,197]. Moreover, inducible systems such as Gal4/UAS or Cre-loxP allow temporal and spatial control of gene expression, facilitating studies on disease onset, progression, and reversibility [55,198]. Beyond elucidating disease mechanisms, these models accelerate the translation of basic research into therapeutic innovations, ultimately guiding the development of targeted treatments and personalized approaches for CNS disorders.

## 4. Conclusions

The increasing complexity of neurological disorders demands experimental models that allow dynamic interactions between molecular, cellular, and systemic levels to be explored in vivo. Zebrafish has emerged not merely as a convenient vertebrate model but as a strategic system that combines genetic manipulability, physiological relevance, and versatility features. Its capacity to recapitulate diverse aspects of central nervous system pathology, ranging from acute injuries to chronic degeneration, highlights its value as a translational bridge between fundamental neurobiology and applied research.

However, the full potential of zebrafish neuroscience has not yet been fully realised. Greater standardisation of experimental paradigms, deeper molecular characterisation of disease phenotypes, and improved alignment with mammalian pathophysiology are needed to enhance its predictive power for human conditions. Rather than replacing traditional models, zebrafish offers an opportunity to complement and expand them, providing unique biological insights and accelerating the development of diagnostic tools and therapies. Yet, fundamental intrinsic limitations constrain the translational utility of zebrafish neuroscience. Differences in brain structural complexity, neural connectivity, and cognitive behaviors, together with gene duplication typical of teleost, may affect the comparability with mammalian systems. These factors necessitate caution while interpreting experimental data and highlight that zebrafish should be considered a complementary model, capable of providing valuable insights but not fully replicating mammalian neurobiology.

## Figures and Tables

**Table 1:** Zebrafish behavioral assays by functional domain and CNS disease relevance.

Behavioural Domain	Assay	Fish Age at Testing (dpf)	Function Assessed	Measured Endpoints	Associated CNS Diseases	Reference
Sensorimotor	Light–dark locomotor assay	Until 6 dpf	Basal locomotion; sensorimotor reactivity	Distance; velocity; activity change at light-dark transitions	Neurodevelopmental toxicity; activity dysregulation	[110]
	Tap stimuli	6 dpf	Startle circuitry; habituation	Startle latency; magnitude; habituation rate	Neurodevelopmental sensorimotor deficits	[98]
	Prepulse inhibition (PPI)	6 dpf	Sensorimotor gating	% inhibition of startle by prepulse	Schizophrenia spectrum	[98]
Anxiety & Exploration	Open field test (OFT)	4–6 months	Anxiety; exploration; locomotion	Total distance; thigmotaxis; time in centre vs. periphery	Anxiety disorders	[95]
	Novel odour exploration test (NOEt)	14 dpf	Chemosensory anxiety/readout	Approach/avoidance to novel odour zone	Anxiety disorders; neophobia	[96]
Memory & Learning	Y-maze memory task (spatial)	<8 months	Spatial learning; working memory	% correct choices; latency; alternation	Alzheimer’s disease; cognitive impairment	[111]
	Novel object recognition (NORt)	7, 14, 21 dpf	Recognition/episodic-like memory	Novelty preference index (novel vs. familiar)	Memory and learning deficits	[94]
	Visual discrimination assay	adult	Associative learning; attention	Accuracy; error rate; learning curve	Sensory and retinal impairments	[112]
Reward/ associative	Conditioned place preference (CPP)	4 to 12 months	Reward/aversion; associative learning	Time spent in conditioned context	Substance use disorders	[97]
Social & Complex Behaviour	Shoaling assay	7, 18, 26, 42, 49, 59, 66, 70, and 76 dpf	Social cohesion/affiliation	Inter-fish distance; shoal area	Autism Spectrum Disorder; schizophrenia (social withdrawal)	[113]
	Conspecific recognition/social preference	adult	Social cognition & memory	Time near conspecifics vs. neutral zone	Social cognition deficits	[114]
	Predator avoidance (fear response)	adult	Threat detection; defensive response	Escape latency & distance	Anxiety-like responses	[115]

**Table 2:** Overview of zebrafish experimental paradigms applied to CNS disorder modelling.

Approach	Experimental Paradigm	Disease Modelled	Phenotype	Reference
Neurotoxic compounds	MPTP (1-methyl-4-phenyl-1,2,3,6-tetrahydropyridine), 6-OHDA, rotenone, paraquat	Parkinson’s disease	Dopaminergic neuron loss via mitochondrial and oxidative stress	[54,120,121]
	Aβ_1–42_ peptides	Alzheimer’s disease	Induced amyloid pathology, apoptosis, cognitive decline	[122]
	Chlorpyrifos (CPF)	Neurodevelopmental toxicity	AChE inhibition, oxidative stress, behavioral alterations	[90,123,124]
	LPS	Neuroinflammation, Alzheimer-like pathology	TLR4 activation, cytokine release	[125,126,127]
	Aluminum chloride + D-galactose	Alzheimer’s-like model	Oxidative stress, amyloid accumulation, aging	[68]
	Heavy metals (Hg, Pb, Cd)	Neurotoxicity	Synaptic disruption, oxidative stress	[128,129,130,131]
Injury models	Spinal cord transection/crush/laser ablation	Spinal cord injury	Neuronal and axonal damage, inflammation, motor deficits	[132,133,134,135,136]
	Brain stab	Traumatic brain injury	Long-lasting motor, cognitive, and behavioral impairments	[137,138]
Morpholino/ mutant lines	PSEN1 morpholino	Alzheimer’s disease	APP/PSEN1 downregulation	[139]
	SOD1; TDP-43; FUS mutant	Amyotrophic lateral sclerosis	Motor neuron degeneration and neuromuscular junction damage	[140]
	Homozygous tpp1 (sa0011) mutant	Neuronal Ceroid Lipofuscinosis type 2	Brain atrophy, and profound neuron loss	[141]
	bace1 mutants (bace1 −/−)	Alzheimer’s disease—Loss of function study	Decreased myelination in peripheral nervous system	[142]
	PARKIN morpholino	Early-onset Parkinson’s disease	Loss of dopaminergic neurons in the posterior tuberculum and mitochondrial complex I dysfunction	[143]
Transgenic lines	Tg (PSEN1)	Early-onset familial Alzheimer’s disease	Dysregulation of mitochondrial energy metabolism, oxidative stress, and altered lysosomal acidification in the brain	[144]
	Tg (LRRK2)	Parkinson’s disease	Impaired mitochondrial homeostasis, dopaminergic neurodegeneration	[145]
	Tg (HuC:Gal4; UAS:TAU-P301L)	Frontotemporal dementia/Tauopathy	TAU hyperphosphorylation, tangle formation, cell death, neuronal and behavioral disturbances	[146]
	PARK7 gene (DJ-1)	DJ-1 loss of function	Genes known to be expressed in (DA) neurons—dopamine transporter (dat; also known as slc6a3), tyrosine hydroxylase (th) and paired-like homeodomain 3 (pitx3)—were also found to be downregulated	[147]

## Data Availability

Not applicable.

## Abbreviations

The following abbreviations are used in this manuscript:

CNS	Central nervous system
GBD	Global Burden of Disease
WHO	World Health Organization
DALY	Disability-Adjusted Life Year
YLL	Years of life lost
YLD	Years lived with disability
BBB	Blood–brain barrier
ALS	Amyotrophic lateral sclerosis
Dm	Dorsomedial zone
Dc	Central zone
Dl	Dorsolateral areas
Dp	Dorsoposterior areas
3Rs	Replacement, Reduction, and Refinement
ZFET	Zebrafish Embryo Toxicity Test
OECD	Organisation for Economic Co-operation and Development
hpf	Hours post fertilization
SCI	Spinal cord injury
TBI	Traumatic brain injury
AD	Alzheimer’s disease
MPTP	1-methyl-4-phenyl-1,2,3,6-tetrahydropyridine
PTZ	Pentylenetetrazole
TH	Tyrosine hydroxylase
SNCA	Human α-synuclein
OPs	Organophosphates
CPF	Chlorpyrifos
AChE	Acetylcholinesterase
NVU	Neurovascular unit
NOEt	Novel odour exploration test
NOR	Novel object recognition
CPP	Conditioned place preference
PPI	Prepulse inhibition
ROS	Reactive oxygen species
Hg	Mercury
Cd	Cadmium
Pb	Lead
LPS	Lipopolysaccharide
TLR4	Toll-like receptor 4
RNS	Reactive nitrogen species
ZO	Zonula occludens
CYP	Cytochrome P450
SrRCs	Stress-responsive regenerating cells
